# Akt regulates neurite growth by phosphorylation-dependent inhibition of radixin proteasomal degradation

**DOI:** 10.1038/s41598-018-20755-w

**Published:** 2018-02-07

**Authors:** Eun-Ju Jin, Hyo Rim Ko, Inwoo Hwang, Byeong-Seong Kim, Jeong-Yun Choi, Kye Won Park, Sung-Woo Cho, Jee-Yin Ahn

**Affiliations:** 1 Department of Molecular Cell Biology, Sungkyunkwan University School of Medicine, Suwon, 16419 Korea; 2 Single Cell Network Research Center, Sungkyunkwan University School of Medicine, Suwon, 16419 Korea; 3 Department of Food Science and Biotechnology, College of Biotechnology and Bioengineering, Sungkyunkwan University, Suwon, 16419 Korea; 4 Department of Biochemistry and Molecular Biology, University of Ulsan College of Medicine, Seoul, 05505 Korea; 5 Samsung Medical Center, Seoul, 06351 Korea

## Abstract

Neurite growth is controlled by a complex molecular signaling network that regulates filamentous actin (F-actin) dynamics at the growth cone. The evolutionarily conserved ezrin, radixin, and moesin family of proteins tether F-actin to the cell membrane when phosphorylated at a conserved threonine residue and modulate neurite outgrowth. Here we show that Akt binds to and phosphorylates a threonine 573 residue on radixin. Akt-mediated phosphorylation protects radixin from ubiquitin-dependent proteasomal degradation, thereby enhancing radixin protein stability, which permits proper neurite outgrowth and growth cone formation. Conversely, the inhibition of Akt kinase or disruption of Akt-dependent phosphorylation reduces the binding affinity of radixin to F-actin as well as lowers radixin protein levels, resulting in decreased neurite outgrowth and growth cone formation. Our findings suggest that Akt signaling regulates neurite outgrowth by stabilizing radixin interactions with F-actin, thus facilitating local F-actin dynamics.

## Introduction

Ezrin, radixin, and moesin, collectively known as ERM proteins, coordinate membrane–cytoskeletal interactions for various forms of cell motility including neuron morphogenesis. ERM proteins share a C-terminal actin-binding domain and an N-terminal FERM domain that binds to membrane proteins such as CD44 and the axon adhesion molecule L1^1,2^, thereby linking filamentous actin (F-actin) and the membrane to regulate growth cone dynamics^3^. ERM proteins also act as scaffolds for adaptor and signaling molecules that regulate cytoskeletal dynamics. The activity of each ERM protein is regulated by the phosphorylation of a conserved threonine residue in the actin-binding domain (T567 in ezrin, T564 in radixin, and T558 in moesin) that blocks the intramolecular association of the N- and C-terminal regions and allows ERM proteins to bind to F-actin and other proteins^4–7^. However, it is not known whether additional phosphorylation on C-terminus of ERM is related to its functions.

In developing neurons, ERM proteins are expressed in growth cones and among ERM proteins, radixin is predominant at the leading edge of dorsal root ganglion (DRG) growth cones^8,9^. In sympathetic neurons, nerve growth factor (NGF) deprivation-induced growth cone collapse is accompanied by a local decrease in radixin levels. The suppressed expression of radixin and moesin, but not of ezrin, impairs growth cone morphology, cytoskeletal organization, and growth cone motility in cortical and hippocampal neurons^10,11^. In addition, the suppression of ERM phosphorylation by inhibiting phosphoinositide 3-kinase (PI-3K) in the growth cones of DRG axons results in growth cone collapse.

The PI-3K pathway regulates diverse neuronal activities, mainly through the downstream molecule Akt/protein kinase B. In addition to a critical role in neuronal survival^12–15^, PI3K/Akt signaling has been implicated in dendritic morphogenesis^16^, neuronal polarity and growth^17^, synaptogenesis and spinogenesis^18^, plasticity^19^, axon establishment, and axon elongation during development by phosphorylating glycogen synthase kinase (GSK)-3β, which leads to GSK3β inactivation^20–23^. While Akt is localized at the axon tip, phosphorylated (inactive) GSK3β is restricted to the tip of growing axons in cultured hippocampal neurons and regulates neuronal polarity. Moreover, Akt links a host of upstream signaling molecules to axon development, axon growth, and dendrite elongation in the central nervous system (CNS) by activating mTORC1 and S6 kinase, which regulate cap-dependent protein translation, and by inhibiting TSC1/2^24–27^. However, evidence suggests that an mTORC1-independent pathway regulates axon regrowth in phosphatase and tensin homolog-deficient neurons^28,29^, causing the aberrant activation of Akt signaling. Moreover, our recent study showed that Akt1 regulates the formation of growth cones and functions through the phosphorylation of S14 on inhibitor of DNA binding 2 (Id2), which is a negative regulator of basic helix–loop–helix transcription factors^30^. Akt-phosphorylated Id2 accumulates at the tip of growing axons where radixin is enriched, and the association with radixin contributes to axon growth and proper growth cone function. Thus, Akt may regulate growth cone dynamics through alternative signaling pathways including the phosphorylation of ERM proteins.

Phosphorylation can promote or inhibit protein ubiquitination in several ways. First, phosphorylation positively or negatively regulates E3 ligase activity through direct phosphorylation on it. For instance, Akt increased E3 ligase activity of Mdm2 by phosphorylation by preventing its autoubiquitination, thereby promotes UPS dependent degradation of p53, which is well known target of Mdm2. In contrast, Mdm2 activity is inhibited by c-Abl phosphorylation of Y394. Second, phosphorylation promotes recognition by an E3 ligase by creating a phosphodegron, short motif that mediate phosphorylation dependent recognition by E3 ligase. The majority of phosphorylation dependent ubiquitination targets are recognized by SCF (Skp1/cullin/ F-box protein) family of E3 ligases, in which the F-box protein associates with the phosphorylated targets^31^. For instance, SCF-beta-TrCP recognizes phosphorylated IkBa or phosphorylated beta-catenin for their ubiquitination. In contrast, instead of promoting ubiquitination, phosphorylation inhibits E3 ligase substrate recognition. For example, Mdm2 binding to p53 is blocked by phosphorylation of S15/T18 in response to DNA damage^32^; S2 phosphorylation in the c-Mos kinase prevents recognition by an unknown E3 ligase^33^. Akt-mediated S143 phosphorylation on DNA methyltransferase-1 (DMNT1) interferes its methylation, which leads to UPS-dependent DNMT1 degradation, inhibiting DNMT1 ubiquitination and enhancing its stability^34^. In addition, Akt-mediated S14 phosphorylation in the Id2 prevents its association with Cdh1/APC/C E3 ligase, thereby stabilizing Id2 protein during neuron development^30^. Third, phosphorylation can influence ubiquitination by resulting substrate/ligase interaction controlling subcellular localization as shown by p27Kip1 nuclear export by its phosphorylation, allowing its degradation by cytoplasmic E3 ligase^35^. Finally, phosphorylation should also regulate deubiquitinating enzyme (DUB) that counteract with E3 ligase. Akt mediated S432 phosphorylation on ubiqutin specific protease (USP) 14, activates its deubiquiting activity^36^. Although it is well established how phosphorylation is important for ubiqutination by E3 ligases and subsequent proteolysis^37^, the regulation of spatial/temporal phosphorylation dependent ubiquitination is understudied and also to what extent phosphorylation can negatively regulate substrate recognition are required further exploration. In the current study, we demonstrated that Akt directly interacts with radixin and phosphorylates T573 rather than T564, which is known to be conserved threonine residue on radixin. Although C-terminal T564 phosphorylation has been well documented by several kinases and conserved throughout many of mammalian radixin, Akt was not the kinase for T564. Akt-mediated phosphorylation of C-terminal T573 on radixin enhances radixin protein stability by preventing proteasomal degradation of radixin. During the differentiation of hippocampal neurons, Akt-mediated radixin phosphorylation is essential for radixin function in the growth cone, including proper neurite outgrowth and F-actin binding. Thus, our data provide additional information on an Akt-dependent pathway for neuron growth regulation through radixin phosphorylation and stabilization.

## Results

### Akt regulates radixin protein stability

**Figure 1:**
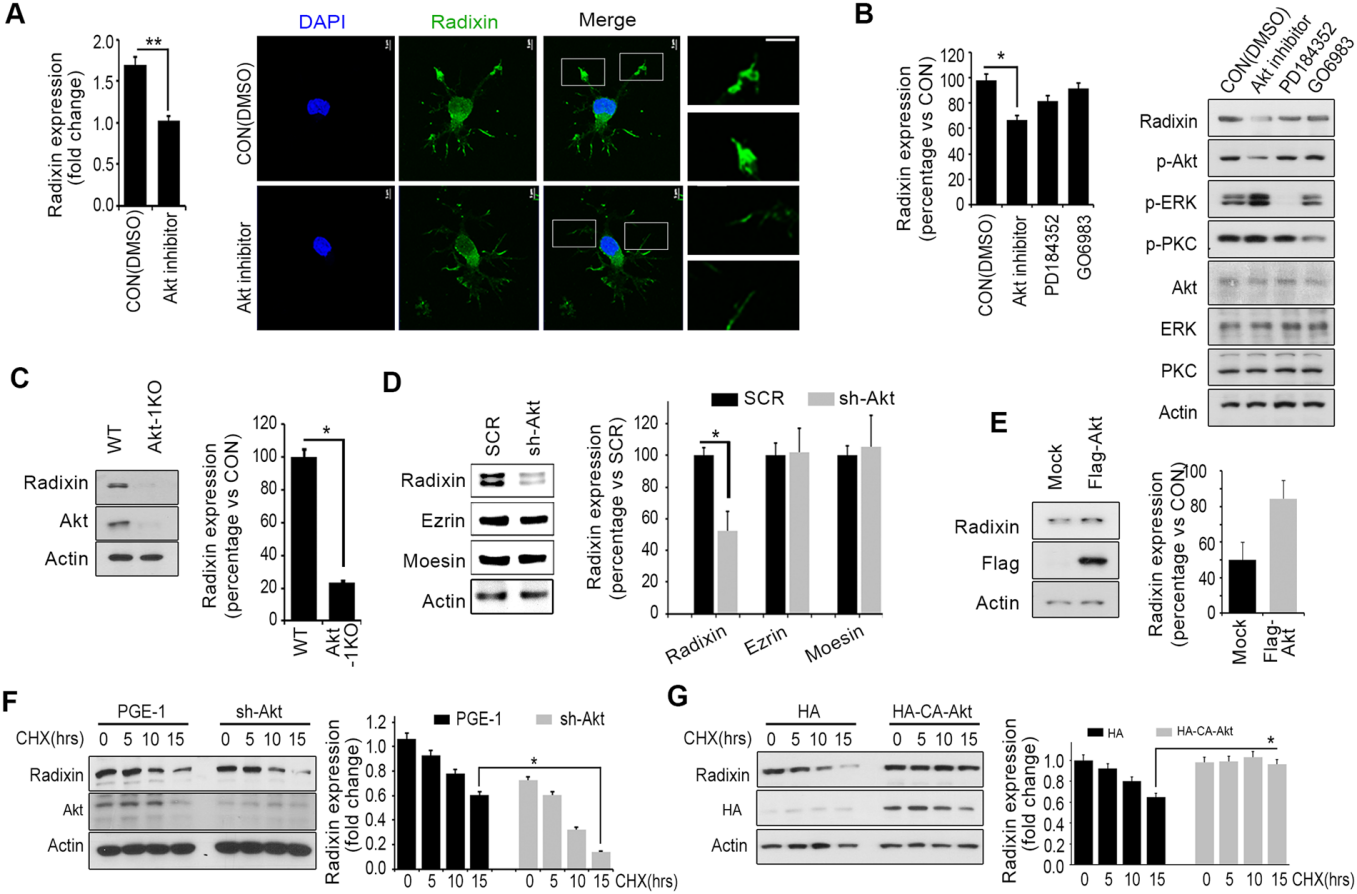
Akt regulates radixin protein stability. (**A**) Hippocampal neurons were treated with a vehicle (DMSO) or Akt inhibitor VIII for 4 h, and radixin expression was examined by immunofluorescence. Enlargement of the boxed area is shown on the right. Scale bar, 50 μm or 5 μm. Enlargement boxes indicate radixin positive filopodia at the axon tip. The bar graph shows radixin immunoreactivity (fluorescence intensity) in the axon. (**B**) PC12 cells were treated with Akt inhibitor VIII (1 μM), ERK/MAPK inhibitor PD184352 (1 μM) or the protein kinase C (PKC) inhibitor GO6983. Amounts of total and phosphorylated Akt, MAPK, and PKC were determined by immunoblotting (IB). *p < 0.05, **p < 0.005 versus indicated. Densitometric values are mean ± SEM from three independent experiments, and the image shown is representative of at least three independent experiments. (**C**) Akt1-KO MEFs were cultured and subjected to immunoblotting analysis with the indicated antibodies. (**D**) PC12 cells were transfected with sh-Akt, and after 48 h, cell lysates were subjected to IB with the indicated antibodies. Densitometric quantification analysis is shown by the bar graph. (**E**) PC12 cells transfected with the flag-Akt or flag control constructs followed by IB. (**F**) Transfected PC12 cells were treated with CHX (20 μM) for the indicated times, and lysates were probed by IB (upper). Knockdown of Akt was confirmed by IB, and radixin protein levels were quantified by densitometry (bottom). (**G**) PC12 cells were transfected with HA-CA (constitutively active form)-Akt or HA-mock vector and treated with cycloheximide (CHX, 20 μM) as indicated time. Half –life of radixin was confirmed by IB and quantification of the radixin protein levels by densitometry analysis (bottom). Values in this figure represent mean ± SEM from three independent experiments and image shown here is representative from at least three independent experiments. The uncropped blot images are shown in Supplementary Fig. 4.

In a previous study, we observed that an Akt1 inhibitor not only impaired the morphology of growth cones with radixin-positive filopodia but also reduced filopodial radixin immunostaining intensity in growing primary hippocampal neurons^30^ (Fig. 1A). We therefore wondered whether Akt kinase affects radixin protein stability. We found that an Akt inhibitor decreased radixin protein levels in PC12 cells as assessed by immunoblotting, while MAPK and protein kinase C inhibitors had little effect (Fig. 1B). Employing Akt1-knock out mouse embryonic fibroblast (MEF) cells, we observed that depletion of Akt1 reduced radixin protein levels (Hereafter Akt indicated Akt1) (Fig. 1C). Moreover, knockdown of Akt using a targeted sh-RNA reduced radixin protein levels while ezrin and moesin levels did not change (Fig. 1D), indicating that Akt specifically regulates radixin protein levels among ERM proteins. Furthermore, Akt overexpression resulted in increased radixin protein levels (Fig. 1E). However, radixin mRNA levels were not altered by either Akt knockdown or overexpression (data not shown) despite the changes in radixin protein levels, indicating that Akt regulates radixin protein levels post translationally. Radixin half-life markedly decreased in Akt-knockdown PC12 cells treated with the eukaryotic protein synthesis inhibitor cycloheximide (CHX) compared to control cells (Fig. 1F). In contrast, radixin half-life was stabilized in PC12 cells expressing constitutively active (CA) Akt (Fig. 1G), suggesting that Akt kinase controls radixin protein stability rather than expression.

### Akt phosphorylates radixin at the C-terminal threonine 573

**Figure 2:**
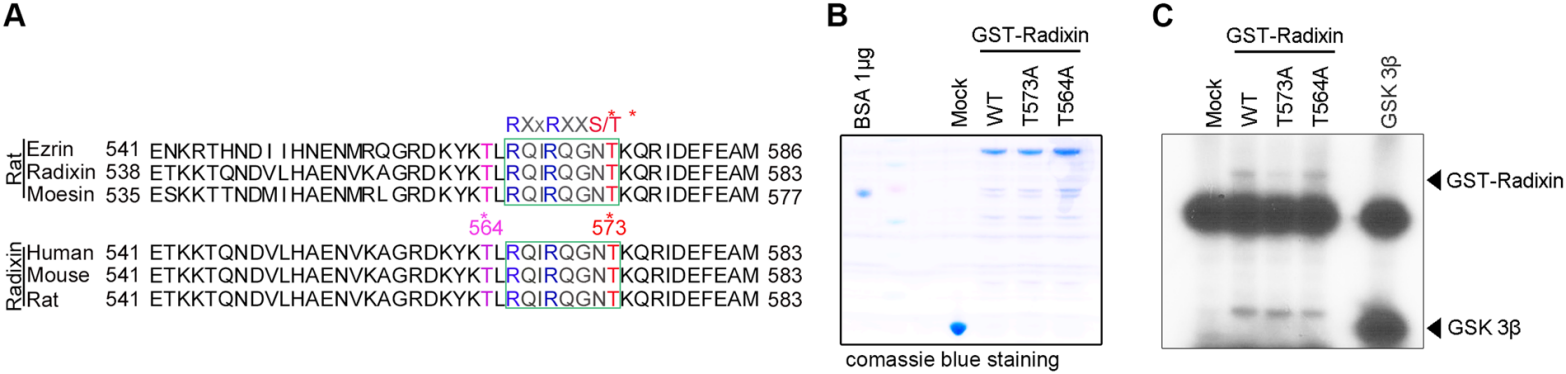
Akt phosphorylates radixin at the C-terminal threonine. (**A**) Comparison of species and rat ERM proteins demonstrate significant homology. ERM proteins share a C-terminal actin-binding domain and an N-terminal FERM domain. The C-terminal residue of a radixin contains a consensus sequence for Akt-mediated phosphorylation. (**B**) Purified bacterial expressing GST-tagged proteins were analyzed by SDS-PAGE followed by coomassie staining. (**C**) An *in vitro* Akt kinase assay was performed with purified active Akt and purified GST fusion proteins of radixin−WT, phosphor-ablation mutant radixin–T573A and T564A. GSK3β fusion and GST proteins were used as positive and negative controls, respectively. Arrows indicate phosphorylated GST-radixin proteins. The image shown is representative of at least three independent experiments. The uncropped blot images are shown in Supplementary Fig 5.

Binding of ERM proteins to F-actin is regulated by phosphorylation at the ERM C-terminus. Multiple kinases have been suggested to phosphorylate the conserved residue in this actin-binding domain of ERM proteins, resulting in ERM protein activation^4–6^. In according to our sequence analysis, we noticed that conserved C-terminal phosphorylation site T564 is not well known putative Akt phosphorylation site while closed T573 is more likely putative Akt phosphorylation site (Fig. 2A). Therefore, to identify specific site for Akt dependent phosphorylation on radixin, we generated and purified GST-tagged wild-type (WT) radixin (GST–radixin-WT) and GST–radixin–T573A, a phosphor-ablation mutant in which T573 putative threonine residue for Akt kinase substrate or GST–radixin–T564A, conserved phosphorylated threonine 564 residue is converted to alanine (Fig. 2B). Our *in vitro* kinase assay using [γ-^32^P] ATP demonstrated that radixin–WT or T564A mutant was substantially phosphorylated by Akt relative to a known positive control substrate, GSK3 fusion protein, whereas radixin–T573A was not, indicating that Akt phosphorylates the T573 of radixin instead conserved threonine 564 residue (Fig. 2C).

### Akt interacts with phosphorylated radixin

**Figure 3:**
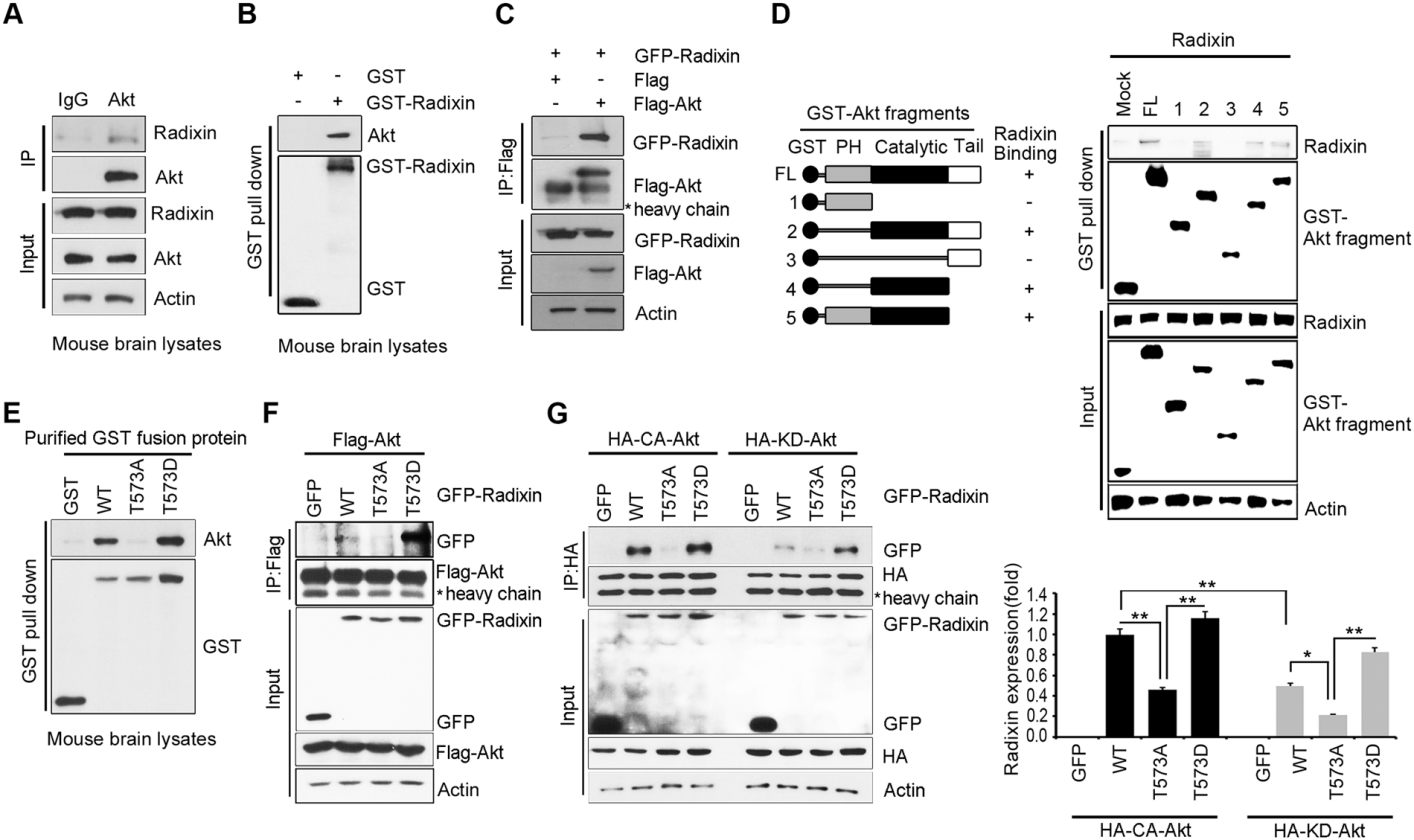
Akt interacts with phosphorylated radixin. (**A**) P7 mouse brain lysates were subjected to immunoprecipitation with anti-Akt antibody or normal IgG. Protein levels were analyzed by anti-Akt and anti-radixin antibodies. (**B**) P7 mouse brain lysates were incubated with purified GST–radixin proteins and GST beads for 3 h at 4 °C with gentle agitation. The interaction between endogenous Akt and purified radixin was examined by IB with anti-Akt antibody. Purified protein expression level was confirmed with anti-GST antibody. (**C**) HEK293T cells were co-transfected with GFP−radixin and Flag–Akt. At 24 h after transfection, cell lysates were subjected to immunoprecipitation with anti-Flag antibody and IB with the indicated antibodies. (**D**) Schematic diagram of the Akt fragments used to determine the binding site (upper). Mammalian GST–Akt fragments were transfected into 293 T cells, and lysates were subjected to GST pull-down assay and IB with anti-radixin antibody (bottom). The expression levels of transfected GST–Akt fragments and endogenous expression of radixin were confirmed by IB. (**E**) *In vitro* binding assay was performed using brain lysates of P7 mouse and purified GST–radixin−WT, GST–radixin–T573D, or GST–radixin–T573A. GST pull-down assay and IB were conducted using the indicated antibodies. (**F**) HEK293T cells were co-transfected with Flag–Akt and GFP−radixin−WT, GFP−radixin–T573A, GFP−radixin–T573D, or GFP (mock), and the cell lysates were subjected to immunoprecipitation with anti-Flag antibody. Protein expression levels were determined by IB using the indicated antibodies. (**G**) HEK293T cells were transfected with HA-tagged CA-Akt or KD-Akt together with GFP−radixin−WT, radixin–T573A, or radixin–T573D. Cell extracts were subjected to immunoprecipitation with anti-HA antibody. Proteins were analyzed by IB with the indicated antibodies. Quantification of radixin protein levels by densitometry is shown on the right. **p < 0.005 versus the indicated treatment group. Values are mean ± SEM from three independent experiments, and the image shown is representative of at least three independent experiments. The uncropped blot images are shown in Supplementary Fig. 6.

We next determined whether radixin physically interacts with Akt. We found an endogenous interaction between Akt and radixin in mouse brain lysates (Fig. 3A). The specific interaction was confirmed in mouse brain extract by using purified GST-radixin protein (Fig. 3B). By employing Flag-tagged Akt and GFP-radixin, the reciprocal immunoprecipitation experiment with anti-GFP antibody produced similar results that demonstrated the association of Akt and radixin (Fig. 3C). An *in vitro* mapping experiments using a series of Akt fragments expressed as GST fusions in HEK 293 cells demonstrated that the catalytic domain of Akt was required for interaction with radixin, supporting the notion that radixin is an Akt kinase substrate (Fig. 3D).

To investigate whether Akt-mediated radixin phosphorylation affects the association of Akt with radixin, we performed an *in vitro* binding assay in mouse brain lysates with purified GST–radixin–WT, GST–radixin–T573A, or GST–radixin–T573D, a phosphor-mimetic mutant. While Akt strongly bound to radixin–WT or radixin–T573D, the interaction between Akt and radixin–T573A was barely detectable (Fig. 3E). In addition, we found that compared with T573A mutant, T564A mutant tightly associated with Akt, supporting the notion that Akt mediated phosphorylation on radixin could occur on T573 residue (Fig. S1). To confirm the interaction of Akt and radixin in cells, we transfected HEK293T cells with GFP tagged radixin–WT, radixin–T573A, or radixin–T573D, along with Flag–Akt. Our immunoprecipitation assay showed that Akt bound more strongly to radixin–T573D than to radixin-WT or radixin–T573A (Fig. 3F). Taken together, these data reflect that radixin phosphorylation is critical for Akt binding.

Next, we tested if Akt activation is required for the interaction with radixin. Constitutively active (CA)−Akt associated with radixin−WT more strongly than kinase dead (KD)–Akt, indicating that Akt activation is necessary for the interaction with radixin (Fig. 3G, first panel lanes 2 and 6). Both CA−Akt and KD−Akt exhibited minimal interaction with radixin−T573A, whereas KD−Akt interacted as strongly with radixin−T573D as CA−Akt, implying that Akt-mediated phosphorylation is a prerequisite for the interaction of radixin with Akt (Fig. 3G, first panel lanes 4 and 8), implying that Akt-mediated radixin phosphorylation was prerequisite for its interaction with Akt. These data indicate that Akt activation enhanced the interaction with radixin, and it appeared that both Akt activation and Akt-mediated radixin phosphorylation were required for their interaction.

### Akt-mediated radixin phosphorylation prevents ubiquitin–proteasome system (UPS)-dependent radixin degradation

**Figure 4:**
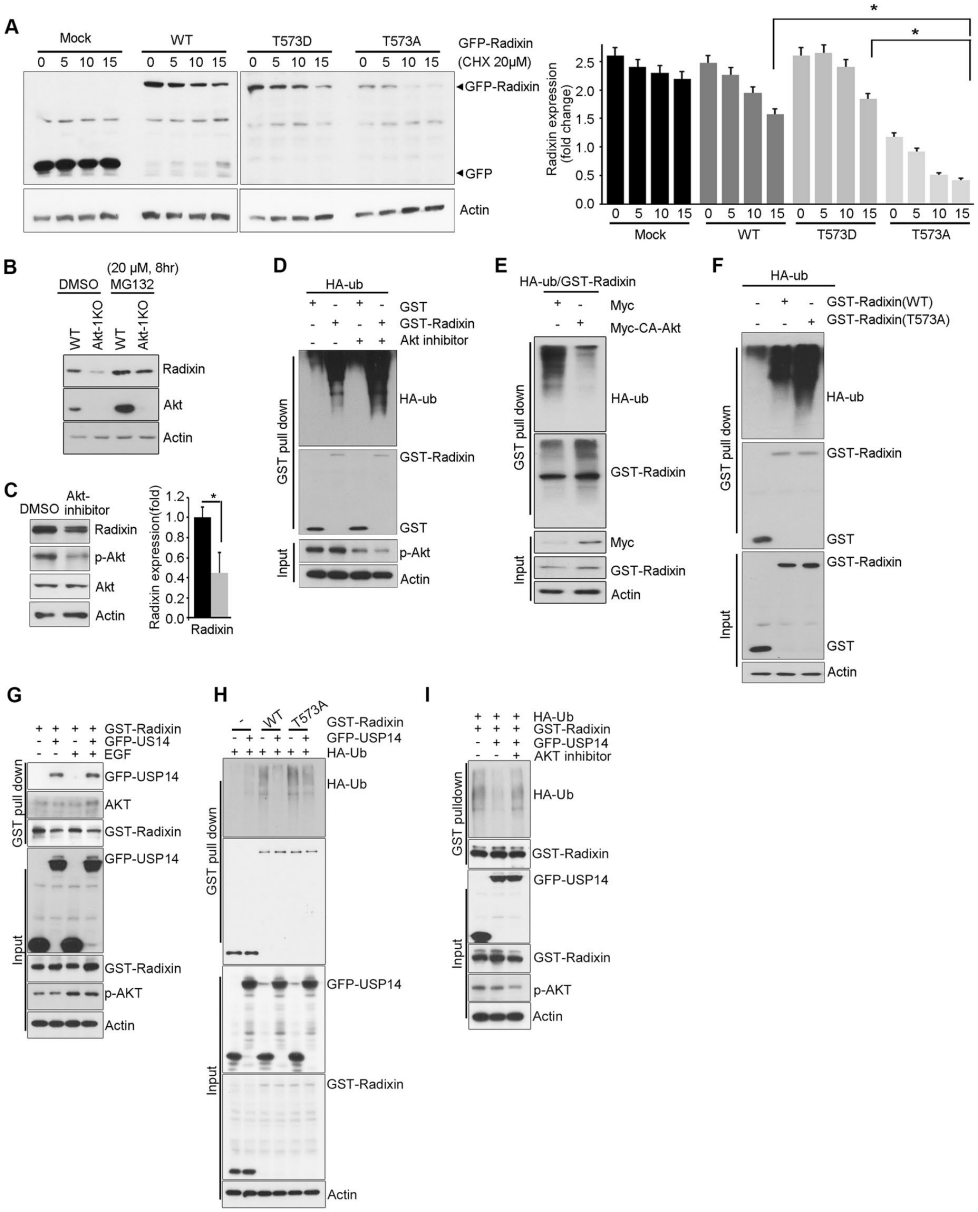
Akt-mediated radixin phosphorylation prevents UPS-dependent radixin degradation. (**A**) PC12 cells were transfected with GFP−radixin−WT, radixin–T573A, or radixin–T573D, and after 24 h treated with CHX (20 μM) as indicated. Lysates were subjected to immunoblotting using anti-GFP antibody. Quantification of radixin protein levels is shown by densitometry (right). *p < 0.05, **p < 0.005 versus indicated. (**B**) Mouse embryonic fibroblasts from Akt1-knockout or WT mice (control) were exposed to 20 μM MG132 (proteasome inhibitor) for 8 h. Cell lysates were subjected to immunoblotting with the indicated antibodies. β-Actin was used as an internal control. The immunoblot shown is representative of at least three independent experiments. (**C**) Hippocampal neurons were treated with a vehicle (DMSO) or AKT inhibitor VIII for 4 h, and lysates were subjected to immunoblotting with the indicated antibodies. Densitometry results are shown on the right. (**D**) PC12 cells were co-transfected with HA−Ub and GST–radixin or GST–vector control and were treated with DMSO or AKT inhibitor VIII for 4 h. Ubiquitination was analyzed with anti-HA antibody. (**E**) PC12 cells were co-transfected with GST-radixin and HA-Ub in the presence of myc control or myc-CA-Akt and ubiquitination was analyzed with anti-HA antibody. (**F**) PC12 cells were co-transfected with HA-Ub and GST-radixin-WT, T573A or GST-vector control. Ubiquitination was analyzed with anti-HA antibody. (**G**) HEK293T cells were co-transfected with GST-radixin and GFP-USP14 or GFP. The serum-starved cells were treated with EGF (50 ng/ml) for 15 minutes. The cell lysates were subjected to GST pulldown assay. Protein expression levels were determined by IB using the indicated antibodies. (**H**) Cells were co-transfected with GST-radixin-WT, T573A and GST along with GFP or GFP-USP14. Ubiquitination was analyzed with anti-HA antibody. (**I**) Cells were co-transfected with GST–radixin and GFP or GFP-USP14 and were treated with DMSO or AKT inhibitor VIII for 4 h. Ubiquitination was analyzed with anti-HA antibody. The uncropped blot images are shown in Supplementary Fig. 7.

Based on our findings that treatment with an Akt inhibitor or Akt knockdown decreased radixin protein levels and that Akt phosphorylates radixin, we investigated whether Akt-mediated phosphorylation contributes to radixin protein stability. The half-life of radixin−T573A was dramatically lower than that of radixin−WT, whereas radixin–T573D was more stable than radixin-WT in PC12 cells (Fig. 4A).

To explore the molecular mechanisms underlying this Akt-mediated enhancement of radixin protein stability, we examined whether proteasomal degradation reduced radixin protein levels in the absence of Akt or Akt inhibition. Pretreatment with the proteasomal inhibitor MG132 protected radixin from degradation in Akt1-knockout mouse embryonic fibroblasts (Fig. 4B). In cultured hippocampal neurons, Akt inhibitor treatment reduced radixin protein levels, concomitant with decreased active Akt (Fig. 4C), suggesting that Akt inhibition decreases radixin protein levels by promoting UPS-dependent degradation and that this might be due to the loss of Akt-dependent phosphorylation. Importantly, radixin was highly ubiquitinated in PC12 cells treated with an Akt inhibitor (Fig. 4D). In contrast, CA-Akt expression blocked radixin ubiquitination (Fig. 4E). Moreover, we observed more abundant ubiquitination of radixin–T573A than of radixin−WT (Fig. 4F). Nevertheless, radixin–T564A mutant exhibited similar levels of ubiquitination with radixin−WT (Fig. S2). Although radixin degradation by the ubiquitin–proteasome pathway has not been reported, recent proteomic studies have suggested that radixin has five putative lysine residues that might be ubiquitinated, although the E3 ligase responsible has not been identified^38–40^. Thus, to the best of our knowledge, our study is the first to reveal that radixin can be degraded by the UPS and that radixin degradation is regulated by Akt-dependent phosphorylation.

Because recent study showed Akt directly phosphorylates S432 on ubiqutin specific protease (USP) 14 and enhances its deubiquiting activity^36^, thereby controlling UPS, we wondered whether Akt activation elicits the connection between radixin T573 phosphorylation and USP14 activation by phosphorylation. We first confirmed that Akt interacts with USP14 (Fig. S3) and examined the interaction between radixin and USP14 using a co-immunopecipitation assay. As shown in Fig. 4G, when radixin and USP14 were overexpressed in HEK293 cells, their interaction was readily detectable and Akt forms tri-complex with radixin and USP14 under epidermal growth factor treatment, which activates Akt, suggesting the possibility radixin is a substrate of USP14 for deubiquitination. To examine whether radixin is a potent substrate for USP14, we conducted ubiquitination assay of radixin. Indeed radixin ubiquitination was reduced when USP14 was co-expressed. However, ubiquitination of radixin–T573A was relatively less affected by USP14 whereas radixin−WT exhibited a deduced level of ubiquitination, suggesting Akt links radixin to USP14 to enhance its stability (Fig. 4H). Treatment of Akt inhibitor greatly increased radixin ubiquitination in the presence of USP14, indicating that Akt enhances protein stability of radixin not only through radixin phosphorylation but also through negatively regulates the UPS recruiting USP14 to radixin (Fig. 4I).

### Disrupting Akt-mediated radixin phosphorylation impairs radixin function in growth cone

**Figure 5:**
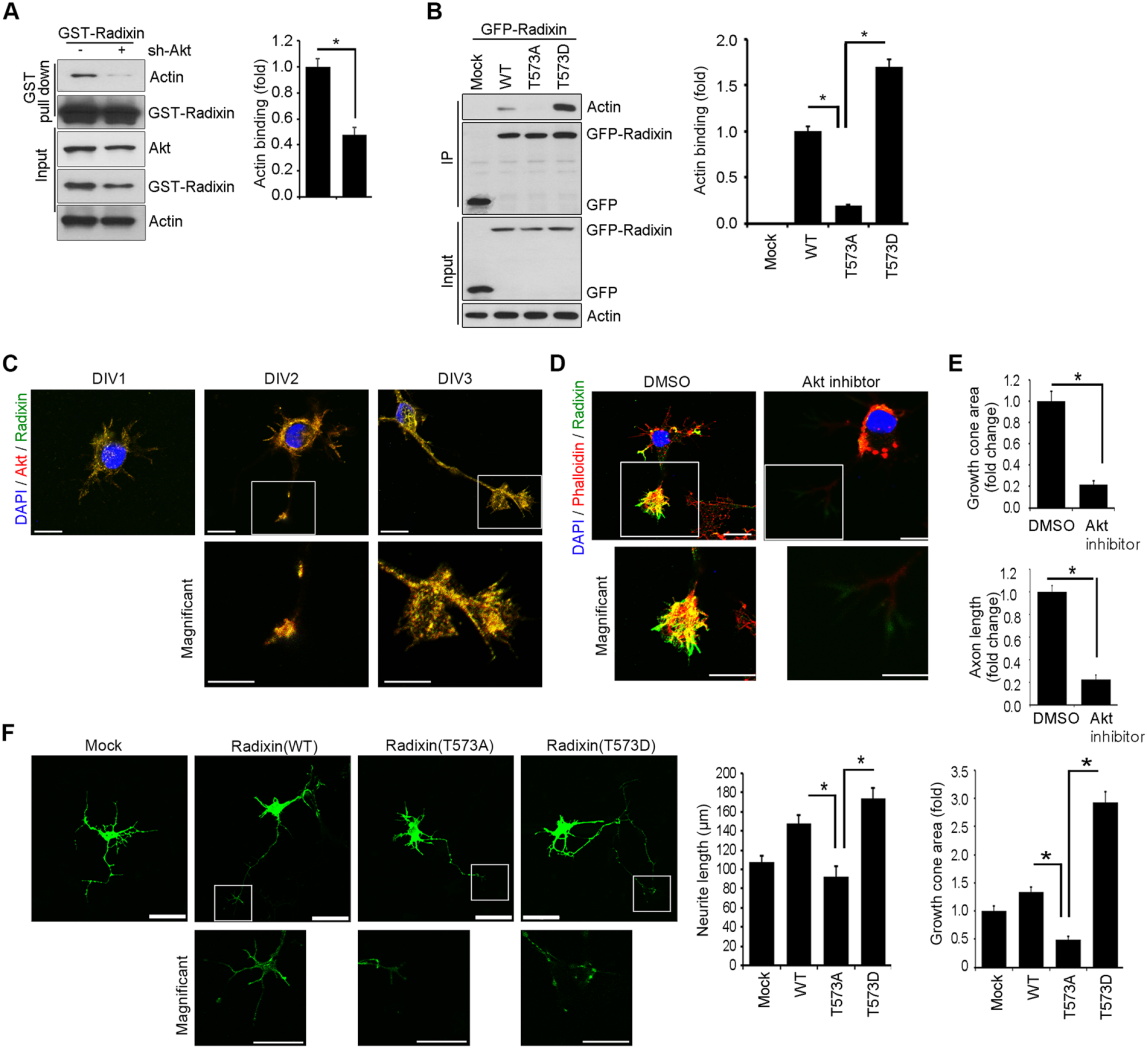
Disrupting Akt-mediated radixin phosphorylation impairs radixin function in growth cone. (**A**) PC12 cells were depleted of Akt by sh-Akt and were transfected with GST–radixin. Lysates were then subjected to GST pull-down assay. Immunoblotting was performed with the indicated antibodies (**B**) Hippocampal neurons (DIV 2) were transfected with GFP−radixin−WT, radixin–T573A, or radixin–573D, and cell lysates were subjected to immunoprecipitation with anti-GFP antibody. Protein-protein interactions are determined by IB using the indicated antibodies. (**A,B**) Actin binding affinity was analyzed by densitometry. *p < 0.05 versus indicated. Values are mean ± SEM from three independent experiments, and the image shown is representative of at least three independent experiments. (**C**) The representative merged image of localization of endogenous Akt (red) and radixin (green) in the developing hippocampus neurons (DIV 1–3). Enlargement of the boxed area is shown on the bottom. Scale bar, 50 μm or 10 μm. (**D**) Cultured hippocampal neurons were exposed to Akt inhibitor VIII and fixed at DIV 3. The neurons stained for radixin (green) and phalloidin (red). Quantification of growth cone size and axon length measurements from three independent experiments is shown (**D,E**). n = 16–24 cells. Error bars, SEM; Scale bar, 10 μm or 50 μm. *p < 0.05 versus indicated. (**F**) Cultured neurons were transfected with GFP fusion proteins of WT radixin, radixin–T573A, radixin–T573D, or GFP vector control at DIV 1 and fixed at DIV 3. The quantification of neurite length from three independent experiments is shown (bottom). **p < 0.005 versus as indicated. Scale bar, 50 μm or 10 μm.

We previously reported that Akt inhibition greatly reduces the immunostaining intensity radixin and the number of radixin-positive filopodia in the growth cones of developing hippocampal neurons^30^. Upon phosphorylation of the conserved C-terminal threonine, radixin binds to F-actin^41^. Thus, we examined the effect of ERM phosphorylation on F-actin binding. The GST pull-down assay showed that the interaction between radixin and F-actin decreased (more than 50%) in the absence of Akt (Fig. 5A). To ascertain functional role of Akt-mediated phosphorylation of radixin in neuron, primary hippocampus neurons were transfected with GFP−radixin−WT, T573A or T573D. While radixin−WT and radixin–T573D bound to F-actin in hippocampal neurons (DIV 3), radixin–T573A exhibited a much weaker interaction (Fig. 5B). Thus, our data indicate that Akt-mediated radixin phosphorylation is critical for its interaction with F-actin, which is required for proper cytoskeletal organization in growth cones.

During the differentiation of hippocampal neurons, Akt co-localized with radixin not only in the cytoplasm but also in growth cone of growing neurites, revealing spatial correlations between Akt and radixin with development of the growth cone (Fig. 5C). To further determine the role of Akt/radixin signaling in F-actin organization in growth cone, we monitored phalloidin-labeled F-actin and radixin expression in the presence of Akt inhibitor VIII during the differentiation of hippocampal neurons. Akt inhibition impaired axon growth, diminishing radixin immunoreactivity and dramatically disrupting growth cone shape, visualized with expression of phalloidin-labeled F-actin (Fig. 5D). Quantitative analysis of neuronal response involving alteration of the growth cone indicated noticeable reduction of growth cone size and shortening of axon length (Fig. 5E). Next, we evaluated the effect of the ablation of T573 phosphorylation on radixin function in the growth cone. We transfected WT, T573A, or T573D radixin into cultured hippocampal neurons and maintained them. Cultured hippocampal neurons at DIV 3 were assessed to determine axon growth and growth cone morphology. Neurons expressing radixin-WT or radixin–T573D developed longer axons and normal architecture of growth cone than vector-expressing control neurons. In contrast, neurons expressing radixin–T573A displayed shorter axons and distorted growth cone shape (Fig. 5F), implying that Akt-mediated phosphorylation is required for radixin−F-actin binding and neuritogenesis regulation in growth cones.

## Discussion

Our study revealed a signal transduction role for radixin as a new kinase substrate for Akt in the developing neuron. Akt directly interacts with and phosphorylates the C-terminal T573 of radixin, required for both F-actin binding and protein stability. Inhibition of Akt or ablation of radixin−T573 phosphorylation disrupted F-actin binding and suppressed neurite outgrowth, suggesting that Akt signaling contributes to maintenance of the normal growth cone structure and neurogenesis through the regulation of radixin protein stability.

Multiple studies have proposed that neurite outgrowth regulation and growth cone formation by Akt are mainly mediated through GSK3β phosphorylation^8,20,42^. However, an Akt-independent function of GSK3β has also been suggested in axon growth and polarization. We demonstrated a novel Akt/Id2-dependent pathway, suggesting that other Akt substrates regulate neurite growth. In the present study, an Akt inhibitor decreased radixin activity and protein levels in developing neurons, prompting us to consider the possibility that Akt controls neurite growth by directly regulating radixin protein levels. NGF induces ERM phosphorylation, and the PI3-K inhibitor LY294002 reduces the NGF-mediated increase in phosphor-ERM staining intensity in DRG neurons^12^. It has also been reported that NGF-induced moesin phosphorylation is mediated by Akt^43^. Akt has not been specifically identified as a radixin kinase. Here we found that Akt phosphorylated the C-terminal T573 of radixin and that T573 phosphorylation was essential for the interaction between radixin and Akt and also between radixin and F-actin in neuronal cells. Although very recent study showed that in WIF-B cells, Akt2 phosphorylated T564 on radixin after insulin stimulation^44^, our *in vitro* kinase assay failed to show that Akt mediated phosphorylation of T564, revealing obvious phosphorylation on T564A mutant form (Fig. 2B), suggesting not only T564 phosphorylation but also other C-terminal phosphorylation including T573 could be required for the activation of radixin.

Akt is belonging to AGC kinase family since they have extensive homology to protein kinase A, G, and C within its kinase domain. It has been well known that Akt phosphorylates its substrate recognizing specific sequence as RXRXXS/T^45^, where X represents any amino acid. R residues (at −5 and −3 positions) are critical requirement for Akt mediated phosphorylation, distinguishing substrate specificity from that of two other mitogen stimulated AGC kinases, RSK (MAPKAP-K1) and S6K1(p70S6K), which can better tolerate K at these position. Although it is possible that there are unknown sequence contexts or macromolecular interactions within cells that might be phosphorylate motifs other than well-agreed motif, currently beyond this category, there are no rigorously demonstrate and independently confirmed Akt substrate. Based on this substrate specificity of Akt kinase, it is not surprising that Akt phosphorylates radixin on T573 (RQIRQGNT) but not T564 (AGRDKYKT) in our study (Fig. 2A). T573 residue is evolutionally well conserved among species implying the role of T573 phosphorylation is important for regulating radixin turn over in the growth cone.

Proper neurite outgrowth and subsequent axon formation depend on a balance between p-ERM−F-actin dynamics at the periphery and microtubule dynamics at the center of filopodia^11,46^. Lamellipodial veils and filopodial extensions are the actin rich sites at which growth cones undergo elongation and/or retraction and are essential for growth cone motility. Among ERM protiens radixin is enriched in lamillipodial veins and filopodial extensions in the growth cones of many types of neuron^10,47,48^. In chick sympathetic neurons, NGF-deprivation induced growth cone collapse was accompanied by reduction of radixin and reintroduction of NGF restored growth cone formation reinstating radixin to these sites. Moreover, radixin is precisely localized towards the leading edges in new direction of growth when growth cone was subjected to an electrical field^10^. Moreover, microscale chromophore assisted laser inactivation of radixin in the growth cone of dorsal root ganglion neurons showed a decrease of lamillipodia in the irradiation region^49^. Furthermore while ezrin and moesin expression is strongest in the central region of growth cone, radixin is highly stained in the peripheral region with phalloidin-label F-actin^9^, indicating specific localization of radixin rather other ezrin or moesin in growth cone might be crucial for normal growth cone morphology and function. In particular our recent study showed that Akt mediated S14 phosphorylation on Id2 dictates its localization at the peripheral region of growth cone, allowing Id2 associates with radixin in the growing axon^30^. Thus, the altered expression of radixin has major effects on cytoskeletal dynamics underlying growth cone activity. Accordingly, Akt inhibition by chemical inhibitor V markedly reduced radixin immunostaining intensity (Fig. 1A), altered growth cone morphology, and suppressed neurite outgrowth (Fig. 5D and E). The expression of radixin–T573A in hippocampal neurons resulted in shorter neurites and lower radixin immunostaining intensity in growth cones than in neurons expressing radixin−WT (Fig. 5F). These data suggest that during neuronal development, Akt phosphorylates radixin and enhances radixin protein stability to regulate proper neurite outgrowth.

Since the ERM proteins possess high sequence similarity of the putative Akt phosphorylation site (T573 in radixin) we cannot rule out the possibility that ezrin and/or moesin might be able to be phosphorylated by Akt. However, in the current study we observed that knockdown of Akt only disturbs radixin protein levels but not ezrin or moesin levels (Fig. 1D). As proper localization and turnover of proteins in growth cone is essential for growth cone function, conceivably, Akt mediated phosphorylation regulates protein turnover of radixin to orchestrate growth cone formation with other proteins such as Id2 in the growing axonal tip as radixin preferentially exists in the peripheral region.On the other hands, although the ERM proteins are functionally redundant when co-expressed in mammalian cells, it might be possible that spatial/temporal expression of ERM proteins in the developing neurons could elicit differential post-translation modification of these proteins including phosphorylation and ubiquitination and/or different interacting partners, thereby leading to have distinct regulatory mechanism. It will be worthy to further investigate how Akt signaling precisely regulate radixin and whether different regulatory mechanism for ezrin or moesin is coordinated with Akt signaling in neuronal development.

This effect of Akt-dependent phosphorylation on radixin protein levels but not on radixin mRNA expression, suggesting the regulation of protein stability, was striking because studies on radixin degradation have not been reported. CHX treatment for up to 8 h did not alter radixin protein levels, indicating that radixin is a relatively stable protein. However, overexpressing radixin–T573A or knocking down Akt markedly decreased radixin protein levels (Figs 1F and 4A), suggesting that this stability is normally maintained by minimal Akt-mediated phosphorylation.

The mechanism by which Akt phosphorylation enhances radixin protein stability remains unclear. Pretreatment with the proteasome inhibitor MG132 protected radixin from degradation under low Akt expression, suggesting that reduced Akt-mediated phosphorylation downregulates radixin by promoting ubiquitin-dependent proteasomal degradation (Fig. 4B). In addition, radixin ubiquitination was elevated by Akt inhibition and reduced by Akt-mediated T573 phosphorylation (Fig. 4D and E). In agreement with our observations, proteomic profiling recently suggested that radixin can be ubiquitinated^38–40,50–52^. However, the E3 ligase has not been identified, and the spatiotemporal profile of radixin ubiquitination during neuronal development remains to be determined. It has been reported that ezrin may be ubiquitinated by the HECT E3 ubiquitin ligase WWP1/Aip5/Tiul1 in epithelial cells, but no proteasomal degradation was observed^53^. Alternatively, we also suggested USP14 as a possible deubiquitinating enzyme for radixin when Akt is activated, as demonstrating the tri-complex of Akt-radixin-USP14 upon growth factor stimulation and notable decrease of radixin ubiquitination with overexpression of USP14 (Fig. 4G–I). Thus Akt regulates radixin protein level not only through its T573 phosphorylation but also by regulating the rate of proteasomal degradation through cooperating with deubiquitinating protein, USP14 as precise control of UPS allows timely and selective degradation of radixin which is essential for its neural development. Studies on the differential modification of ERM proteins and ensuing effects on expression and function during neuronal development are clearly warranted to fully elucidate the functions of these proteins in neuronal differentiation, circuit formation, and plasticity.

**Figure 6:**
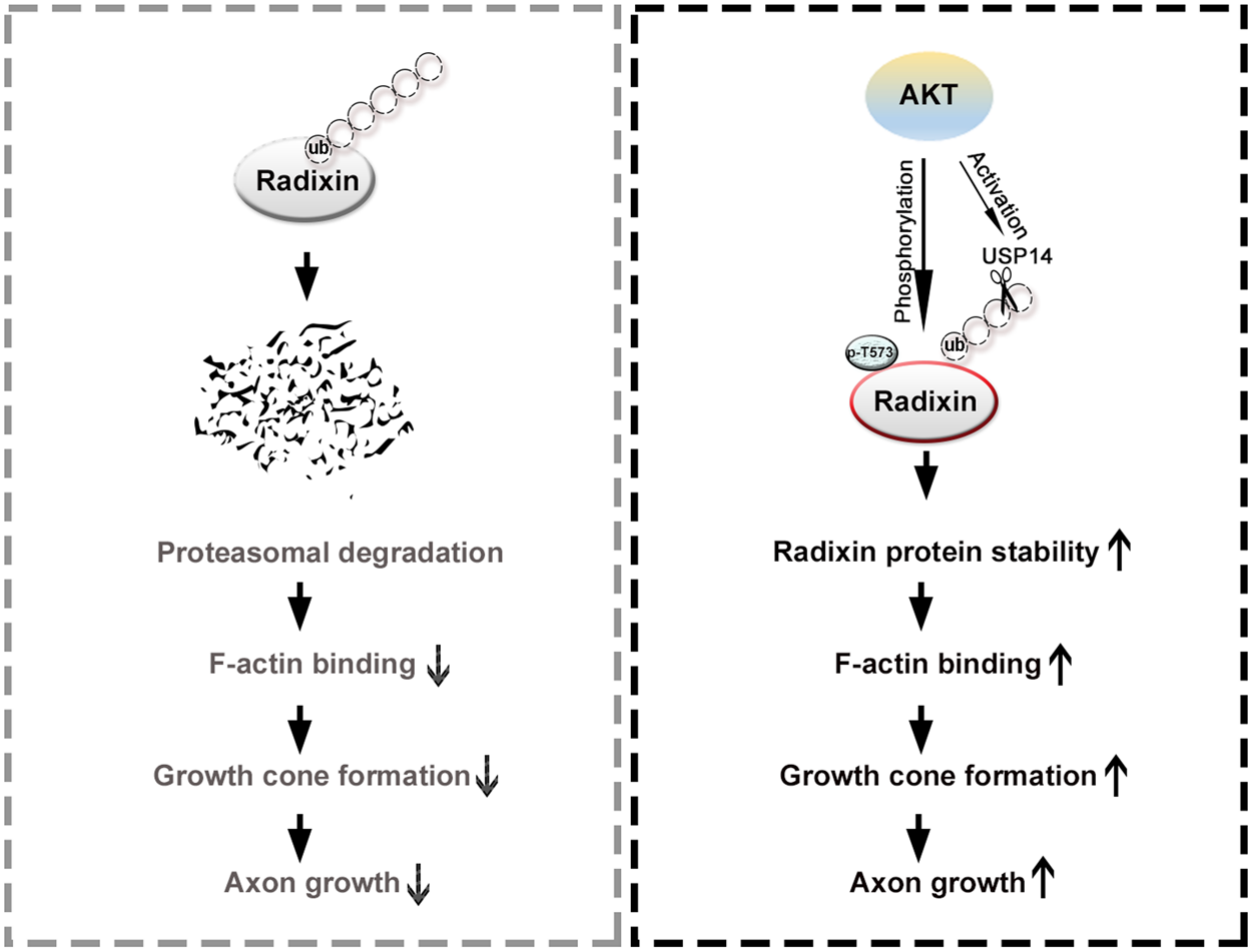
Schematic diagram of Akt/radixin signaling in neurons. Akt-mediated phosphorylation of radixin at T573 augments protein stability and radixin interaction with F-actin, thereby promoting neurite growth in developing neurons.

In summary (Fig. 6), our study establishes a causal connection between Akt and F-actin organization through the modulation of radixin protein stability. We propose that radixin is a novel substrate and binding partner of Akt and that Akt-mediated radixin phosphorylation is an important regulator of intrinsic neurite growth in developing neurons. The regulation of radixin protein stability by Akt signaling may provide a mechanistic basis for understanding the contributions of radixin−F-actin dynamics to neural development.

## Materials and Methods

### Preparation of primary neurons and cell culture

The brains of E18 rat embryos were dissected, and hippocampi were removed and placed in a 15 ml tube with 14 ml of Hanks’ balanced salt solution on ice. The medium was carefully aspirated, leaving 2 ml of the medium in the tube. Papain (20 mg/ml in a dissection medium) was added, and the hippocampi were incubated for 20 min at 37 °C. Digestion was stopped by washing the hippocampi twice with 4 ml of complete medium supplemented with 10% fetal bovine serum (FBS). Then, 3 ml of neurobasal medium (NB, Invitrogen 21103-049)/B27 (Invitrogen 17504-044) was added, and tissues were dissociated by gently triturating the hippocampi through a fire-polished Pasteur pipette. The cell mixture was diluted to 10 ml with NB/B27 and was then filtered through a 40 or 70 µM strainer. Cells were spun at 1800 rpm for 5 min and resuspended in 10 ml of NB/B27. HEK293T and PC12 cells were cultured as previously described^54^. HEK293T cells were cultured in Dulbecco’s modified Eagle’s medium (DMEM) supplemented with 10% FBS and 100 U/ml of penicillin–streptomycin. PC12 cells were maintained in DMEM with 10% FBS, 5% horse serum, and 100 U/ml of penicillin–streptomycin at 37 °C under a 5% CO_2_ atmosphere.

### Antibodies, plasmids, and chemicals

Anti-radixin, and anti-Akt antibodies were obtained from Cell Signaling Technology (Danvers, MA, USA). Anti-GFP, anti-GST, anti-HA, and anti-actin antibodies were acquired from Santa Cruz Biotechnology (Santa Cruz, CA, USA). Alexa Fluor 594-labeled goat anti-rabbit and Alexa Fluor 488-labeled goat anti-mouse secondary antibodies were obtained from Molecular Probes (Eugene, OR, USA). All other chemicals were obtained from Sigma (St. Louis, MO, USA).

### Construction of recombinant DNA

Rat WT radixin cDNA was cloned into a pEGFP vector and amplified using a forward primer beginning at the 5′ Kpn1 site (5′-AAA GGT ACC GCA TGC CGA AAC CAA TAA AT–3′) and a reverse primer containing the BamH1 site at the 3′ end of the gene (3′-CTC AAA CTT CGT TAC ACT CCT AGG AAA-5′). The radixin point mutation T573A was generated by RT-PCR amplification using an *in vitro* site-directed mutagenesis system and was inserted into a pEGFP vector. Primers for T573A (5′-CAA GGC AAC GCC AAG CAG CGC ATC GAT GAG-3′ and 3′-GCG CTG CTT GGC GTT GCC TTG TCG AAT CTG-5′) were synthesized by Cosmo Genetech (Seoul, Korea). WT and mutant radixin were subcloned into pGEX4T-1

### Co-immunoprecipitation and *in vitro* binding assays

For co-immunoprecipitation, cells were rinsed with phosphate-buffered saline (PBS) and lysed in a buffer containing 50 mM Tris-Cl (pH 7.4), 150 mM NaCl, 1 mM EDTA, 0.5% Triton X-100, 1.5 mM Na_3_VO_4_, 50 mM sodium fluoride, 10 mM sodium pyrophosphate, 10 mM beta-glycerophosphate, 1 mM phenylmethylsulfonyl fluoride, and a protease inhibitor cocktail (Calbiochem, San Diego, CA). Cell lysates (0.5 to 1 mg of protein) were mixed with a primary antibody and protein A/G beads and were then incubated for 3 h at 4 °C with gentle agitation. Beads were then washed in a lysis buffer. The extracted proteins were analyzed by immunoblotting as previously described^55^. For GST pull-down assays, cells were rinsed with PBS and lysed in the buffer as previously described^56,57^. Cell lysates (0.5 to 1 mg of protein) were mixed with glutathione–sepharose beads and incubated for 3 h at 4 °C with gentle agitation. The beads were then washed in the lysis buffer, mixed with 2× sodium dodecyl sulfate (SDS) sample buffer, and boiled. Extracted proteins were analyzed by immunoblotting.

### Immunofluorescence

Immunostaining was performed as previously described^58,59^ with the following modifications. Cells grown on coverslips in 24-well plates were fixed in 4% paraformaldehyde for 15 min, permeabilized in PBS containing 0.25% Triton X-100 for 10 min, and blocked in 1% bovine serum albumin for 30 min. Cells were immunostained using primary antibodies and the appropriate Alexa Fluor 594-labeled goat anti-rabbit and Alexa Fluor 488-labeled goat anti-mouse secondary antibodies. Nuclei were counterstained with DAPI. Images of immunostained cells were acquired using a laser scanning confocal microscope (LSM 710, Carl Zeiss, Germany) controlled by ZEN software.

### *In vitro* kinase assay

The *in vitro* kinase assay was performed as previously described^54^. Recombinant active Akt (Upstate Biotechnology, Lake Placid, NY, USA) was incubated with 1.8 × 10^5^ Bq γ-32P-ATP and 1 µg of recombinant GST fusion protein in 30 µl of kinase buffer (25 mM HEPES, 5 mM β-glycerophosphate, 10 mM MgCl_2_, 2 mM dithiothreitol, 0.1 mM NaVO_3_, and 200 µM ATP). Reactions were run at 30 °C for 20 min and terminated by the addition of Laemmli SDS sample dilution buffer. Proteins were separated by 10% SDS-polyacrylamide gel electrophoresis, and phosphorylation was visualized by performing autoradiography.

### Statistical Analysis

Data are expressed as mean ± SEM of three independent experiments with triplicate measurements. Statistical analysis was performed using SigmaPlot (Systat Software, San Jose, CA, USA). All studies were performed in a blinded manner. Statistical significance was defined by Student’s t-test (^*^p < 0.05; ^**^p < 0.005).

### Animal experiments

This study was reviewed and approved by the Institutional Animal Care and Use Committee (IACUC) of Sungkyunkwan University School of Medicine (SUSM) (code16-21/16-22). SUSM is an Association for Assessment and Accreditation of Laboratory Animal Care International (AAALAC International; No. 001004) accredited facility and abide by the Institute of Laboratory Animal Resources (ILAR) guide. All experimental procedures were carried out in accordance with the regulations of the IACUC guideline of Sungkyunkwan University.

## Electronic supplementary material

Supplementary information
