# Supplementary material for: Akt regulates neurite growth by phosphorylation-dependent inhibition of radixin proteasomal degradation
Source: Sci Rep. 2018 Feb 7;8:2557. doi: 10.1038/s41598-018-20755-w (PMC5803261; doi:10.1038/s41598-018-20755-w)
Supplement: Supplementary file 1 — Supplementary information [file 41598_2018_20755_MOESM1_ESM.pdf]

Supplementary Information

Akt regulates neurite growth by phosphorylation-dependent inhibition of radixin proteasomal degradation

Eun-Ju Jin[1,2#], Hyo Rim Ko[1,2#], Inwoo Hwang[1,2], Byeong-Seong Kim[1], Jeong-Yun Choi[1], Kye Won Park[3], Sung-Woo Cho[4], and Jee-Yin Ahn[1,2,5]*

[1]Department of Molecular Cell Biology, Sungkyunkwan University School of Medicine, Suwon 16419, Korea

[2]Single Cell Network Research Center, Sungkyunkwan University School of Medicine, Suwon 16419, Korea

[3]Department of Food Science and Biotechnology, College of Biotechnology and Bioengineering, Sungkyunkwan University, Suwon 16419, Korea

[4]Department of Biochemistry and Molecular Biology, University of Ulsan, College of Medicine, Seoul 05505, Korea

[5]Samsung Medical Center, Seoul 06351, Korea

[#] These authors are equally contributed.

***Correspondence should be addressed to** Jee-Yin Ahn, Department of Molecular Cell Biology, Sungkyunkwan University School of Medicine, 2066, Seobu-ro, Jangan-gu, Suwon 16419, Korea Phone: 82-31-299-6134; Fax: 82-31-299-6139; E-mail: jeeahn@skku.edu

Supplementary Figure legends

Figure S1.

HEK293T cells were co-transfected with Flag–Akt and GFP–radixin–WT, GFP–radixin–T573A, GFP–radixin–T564A, or GFP (mock), and the cell lysates were subjected to immunoprecipitation with anti-Flag antibody. Protein expression levels were determined by IB using the indicated antibodies. The uncropped blot images are shown in supplementary figure 8.

Figure S2.

PC12 cells were co-transfected with HA-Ub and GST-radixin-WT, GST-radixin-T573A, or GST-radixin-T564A and the cell lysates were subjected to GST pull down. Ubiquitination was analyzed with anti-HA antibody. The uncropped blot images are shown in supplementary figure 9.

Figure S3.

(**A**) HEK293T cells were co-transfected with GST-Akt and GFP–USP14, or GFP (mock). The cell lysates were subjected to GST pulldown assay. (**B**) HEK293T cells were co-transfected with GST–radixin and GFP-USP14. At 24 h after transfection, cell lysates were subjected to GST pulldown assay and IB with the indicated antibodies. The uncropped blot images are shown in supplementary figure 10.

Figure S4.

These are uncropped blot images corresponding to data show in Figure 1.

Figure S5.

These are uncropped blot images corresponding to data show in Figure 2.

Figure S6.

These are uncropped blot images corresponding to data show in Figure 3.

Figure S7.

These are uncropped blot images corresponding to data show in Figure 4.

Figure S8.

These are uncropped blot images corresponding to data show in Figure S1.

Figure S9.

These are uncropped blot images corresponding to data show in Figure S2.

Figure S10.

These are uncropped blot images corresponding to data show in Figure S3.

Figure S1

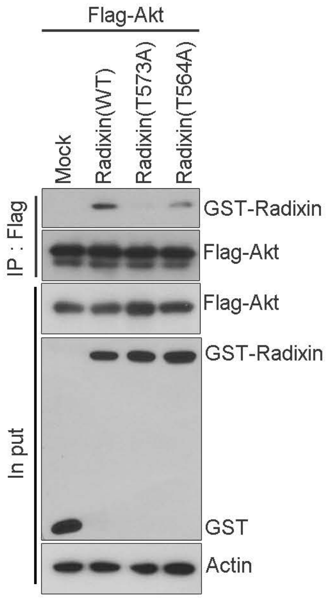

Flag-Akt
Mock
Radixin(WT)
Radixin(T573A)
Radixin(T564A)
IP : Flag
GST-Radixin
Flag-Akt
In put
Flag-Akt
GST-Radixin
GST
Actin

Figure S2

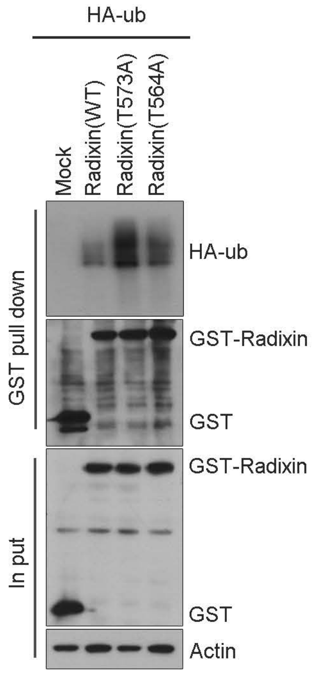
HA-ub
Mock
Radixin(WT)
Radixin(T573A)
Radixin(T564A)
GST pull down
HA-ub
GST-Radixin
GST
In put
GST-Radixin
GST
Actin

Figure S3

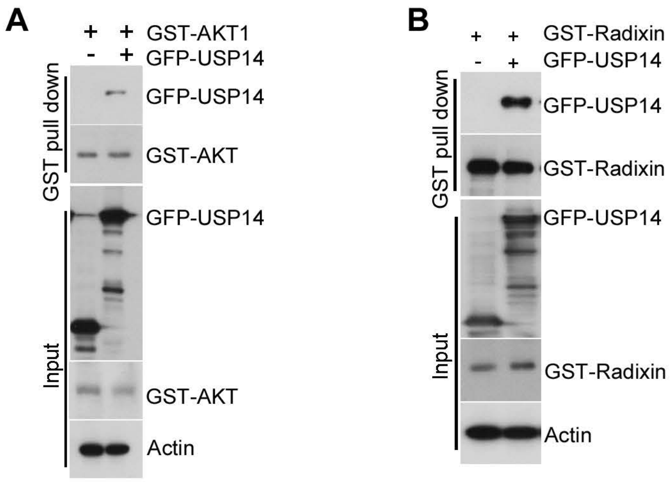
A
+ + GST-AKT1
- + GFP-USP14
GST pull down
GFP-USP14
GST-AKT
Input
GFP-USP14
GST-AKT
Actin
B
+ + GST-Radixin
- + GFP-USP14
GST pull down
GFP-USP14
GST-Radixin
Input
GFP-USP14
GST-Radixin
Actin

Figure S4

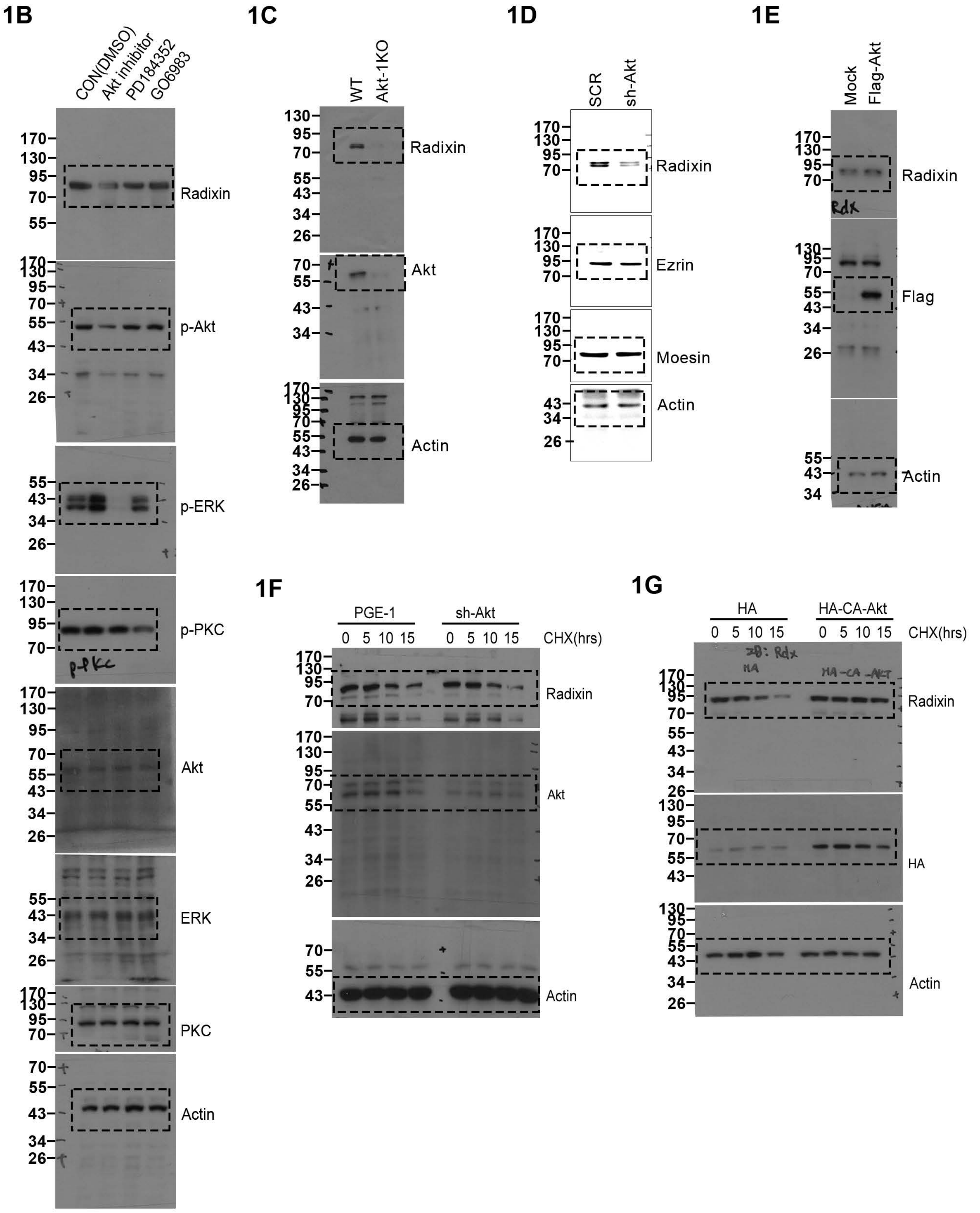

1B
CON(DMSO)
Akt inhibitor
PD184352
GO6983
Radixin
p-Akt
p-ERK
p-PKC
Akt
ERK
PKC
Actin
1C
WT
Akt-1KO
Radixin
Akt
Actin
1D
SCR
sh-Akt
Radixin
Ezrin
Moesin
Actin
1E
Mock
Flag-Akt
Radixin
Flag
Actin
1F
PGE-1
sh-Akt
0 5 10 15 0 5 10 15 CHX(hrs)
Radixin
Akt
Actin
1G
HA
HA-CA-Akt
0 5 10 15 0 5 10 15 CHX(hrs)
Radixin
HA
Actin

Figure S5

2A

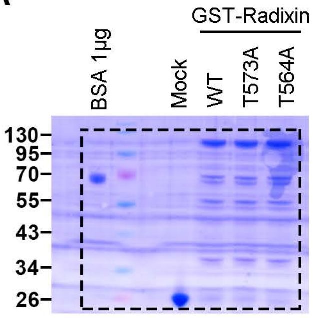

2B

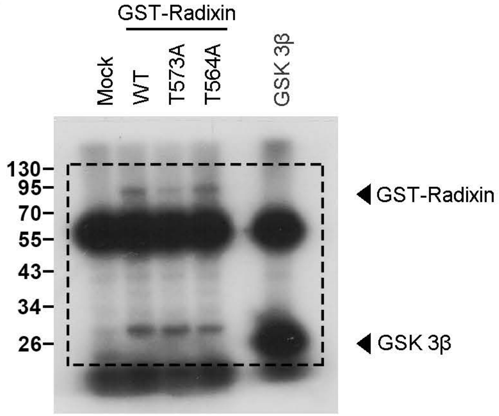

Figure S6

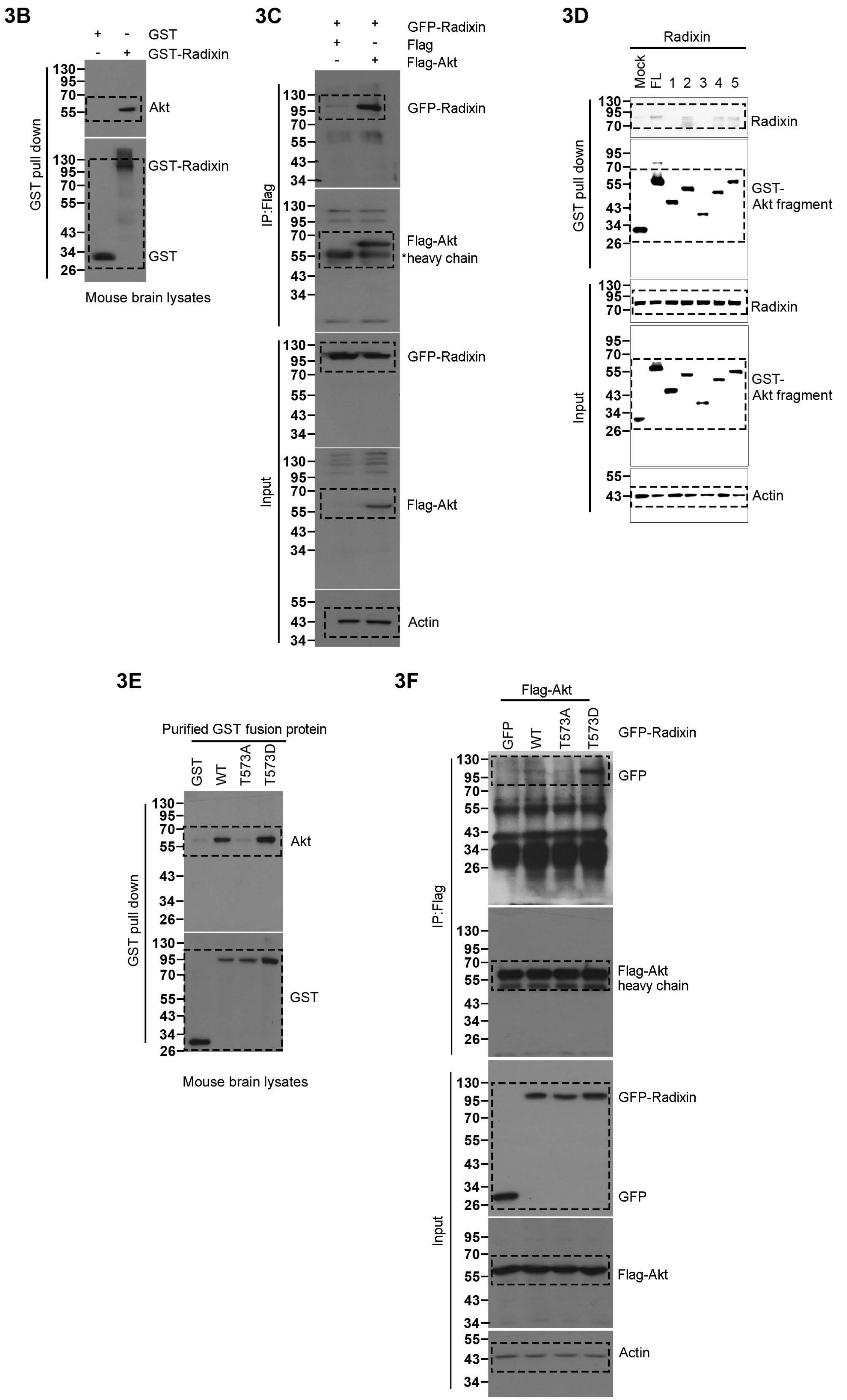

3B
GST
GST-Radixin
GST pull down
Akt
GST-Radixin
GST
Mouse brain lysates
130
95
70
55
43
34
26
3C
GFP-Radixin
Flag
Flag-Akt
IP:Flag
Input
GFP-Radixin
Flag-Akt
*heavy chain
Actin
3D
Radixin
Mock
FL
1
2
3
4
5
GST pull down
Input
Radixin
GST-
Akt fragment
Actin
3E
Purified GST fusion protein
GST
WT
T573A
T573D
GST pull down
Akt
GST
Mouse brain lysates
3F
Flag-Akt
GFP
WT
T573A
T573D
GFP-Radixin
IP:Flag
Input
GFP
Flag-Akt
heavy chain
GFP-Radixin
GFP
Flag-Akt
Actin

Figure S7

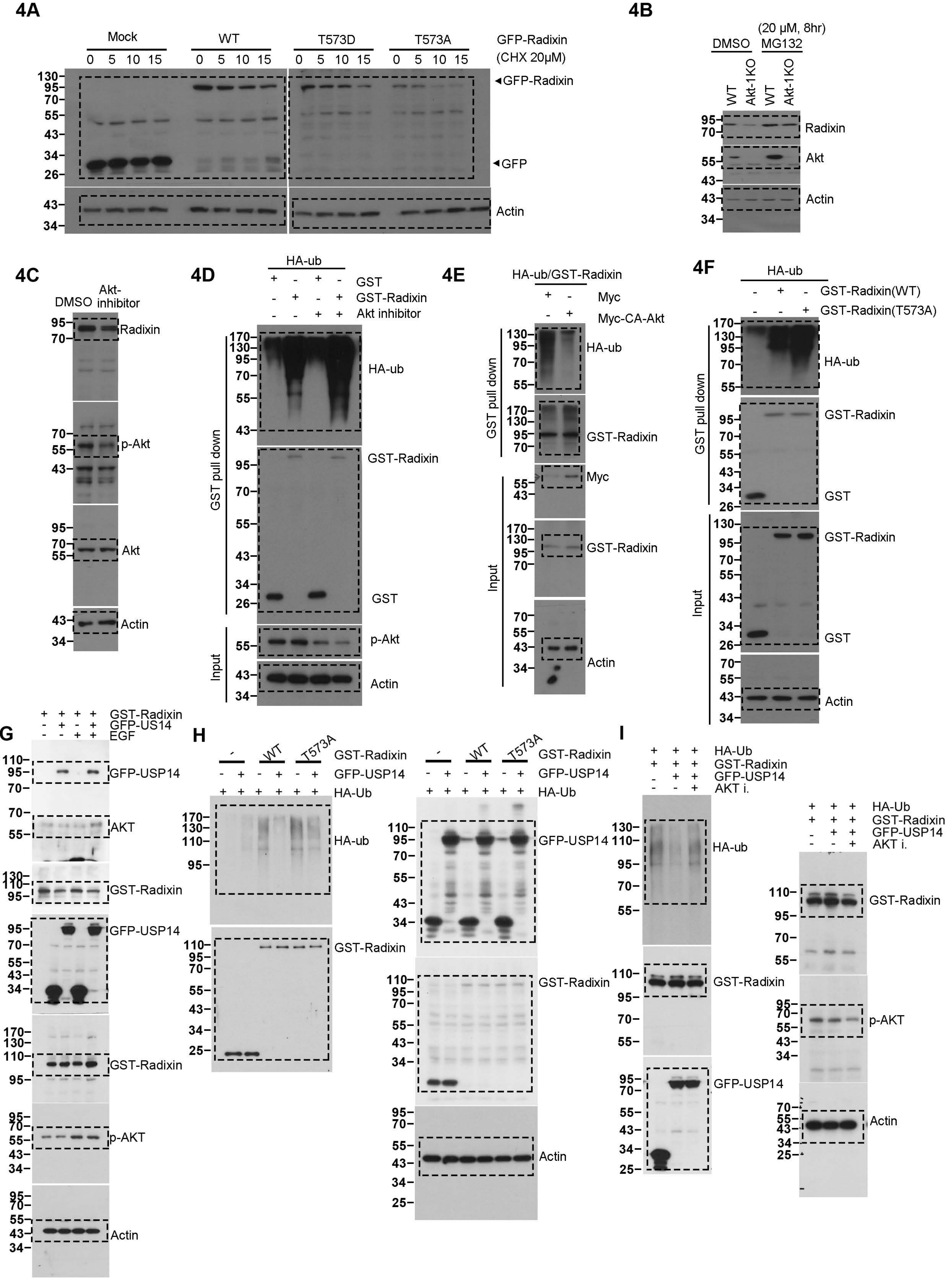
4A
Mock
WT
T573D
T573A
0 5 10 15
GFP-Radixin
(CHX 20μM)
GFP-Radixin
GFP
Actin
4B
DMSO
(20 μM, 8hr)
MG132
WT
Akt-1KO
Radixin
Akt
Actin
4C
DMSO
Akt-inhibitor
Radixin
p-Akt
Akt
Actin
4D
HA-ub
GST
GST-Radixin
Akt inhibitor
GST pull down
Input
HA-ub
GST-Radixin
GST
p-Akt
Actin
4E
HA-ub/GST-Radixin
Myc
Myc-CA-Akt
GST pull down
Input
HA-ub
GST-Radixin
Myc
GST-Radixin
Actin
4F
HA-ub
GST-Radixin(WT)
GST-Radixin(T573A)
GST pull down
Input
HA-ub
GST-Radixin
GST
GST-Radixin
GST
Actin
G
GST-Radixin
GFP-US14
EGF
GFP-USP14
AKT
GST-Radixin
GFP-USP14
GST-Radixin
p-AKT
Actin
H
WT
T573A
GST-Radixin
GFP-USP14
HA-Ub
HA-ub
GST-Radixin
GFP-USP14
GST-Radixin
Actin
I
HA-Ub
GST-Radixin
GFP-USP14
AKT i.
HA-ub
GST-Radixin
GFP-USP14
GST-Radixin
p-AKT
Actin

Figure S8

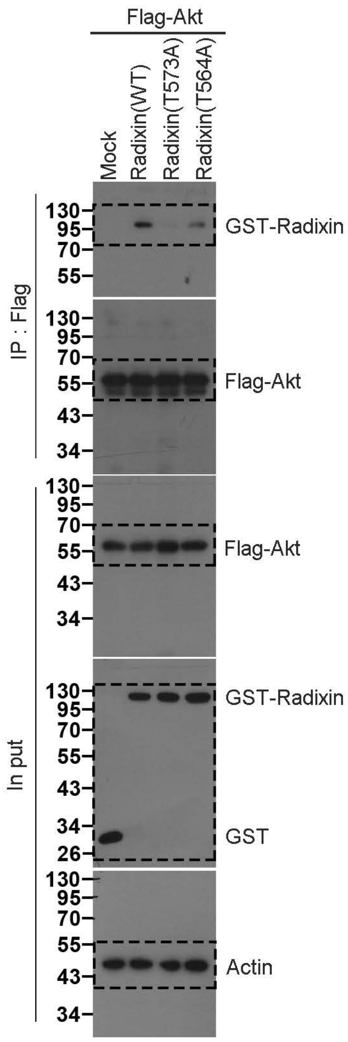
Flag-Akt
Mock
Radixin(WT)
Radixin(T573A)
Radixin(T564A)
IP : Flag
In put
130
95
70
55
43
34
26
GST-Radixin
Flag-Akt
GST
Actin

Figure S9

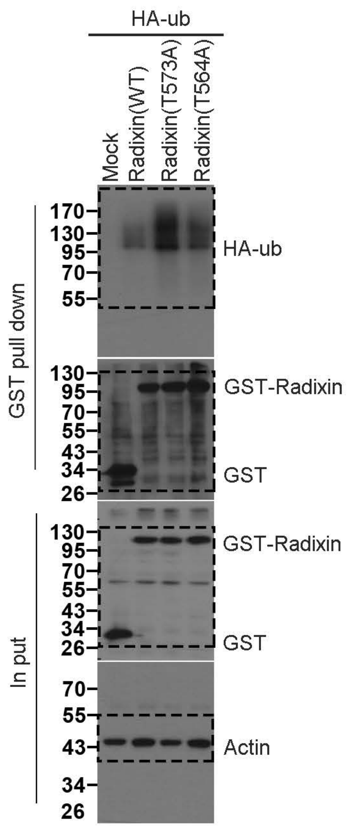
HA-ub
Mock
Radixin(WT)
Radixin(T573A)
Radixin(T564A)
GST pull down
170
130
95
70
55
HA-ub
130
95
70
55
43
34
26
GST-Radixin
GST
In put
130
95
70
55
43
34
26
GST-Radixin
GST
70
55
43
34
26
Actin

Figure S10

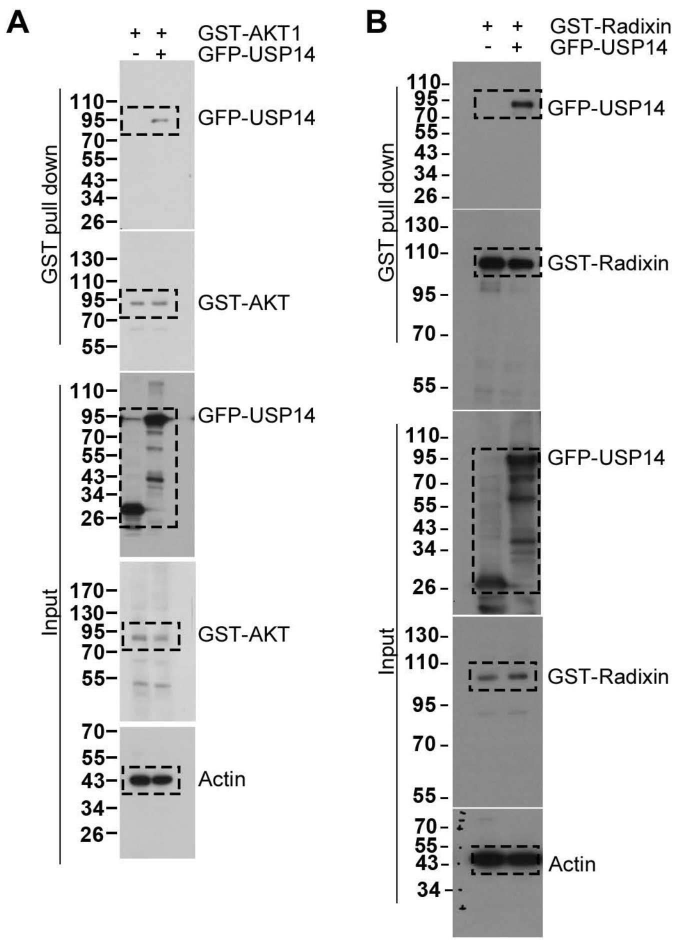
A
GST-AKT1
GFP-USP14
GST pull down
GFP-USP14
GST-AKT
Input
GFP-USP14
GST-AKT
Actin
B
GST-Radixin
GFP-USP14
GST pull down
GFP-USP14
GST-Radixin
Input
GFP-USP14
GST-Radixin
Actin
